# Fisetin, a Natural Polyphenol, Ameliorates Endometriosis Modulating Mast Cells Derived NLRP-3 Inflammasome Pathway and Oxidative Stress

**DOI:** 10.3390/ijms24065076

**Published:** 2023-03-07

**Authors:** Alessia Arangia, Ylenia Marino, Roberta Fusco, Rosalba Siracusa, Marika Cordaro, Ramona D’Amico, Francesco Macrì, Emanuela Raffone, Daniela Impellizzeri, Salvatore Cuzzocrea, Rosanna Di Paola

**Affiliations:** 1 Department of Chemical, Biological, Pharmaceutical and Environmental Sciences, University of Messina, 98166 Messina, Italy; 2 Department of Biomedical, Dental and Morphological and Functional Imaging, University of Messina, Consolare Valeria, 98100 Messina, Italy; 3 Department of Veterinary Sciences, University of Messina, 98168 Messina, Italy,; 4 Department of Maternal and Child Obstetrics and Gynecology, Papardo Hospital, 98166 Messina, Italy; 5 Department of Pharmacological and Physiological Science, Saint Louis University School of Medicine, St. Louis, MO 63104, USA

**Keywords:** antioxidant, inflammation, NLRP3, mast cells, oxidative stress

## Abstract

A chronic, painful, and inflammatory condition known as endometriosis is defined by the extra-uterine development of endometrial tissue. The aim of this study was to evaluate the beneficial effects of fisetin, a naturally occurring polyphenol that is frequently present in a variety of fruits and vegetables. Uterine fragments were injected intraperitoneally to cause endometriosis, and fisetin was given orally every day. At 14 days of treatment, laparotomy was performed, and the endometrial implants and peritoneal fluids were collected for histological, biochemical, and molecular analyses. Rats subjected to endometriosis presented important macroscopic and microscopic changes, increased mast cell (MC) infiltration, and fibrosis. Fisetin treatment reduced endometriotic implant area, diameter, and volumes, as well as histological alterations, neutrophil infiltration, cytokines release, the number of MCs together with the expression of chymase and tryptase, and diminished α smooth muscle actin (α-sma) and transforming growth factor beta (TGF β) expressions. In addition, fisetin was able to reduce markers of oxidative stress as well as nitrotyrosine and Poly ADP ribose expressions and increase apoptosis in endometrial lesions. In conclusion, fisetin could represent a new therapeutic strategy to control endometriosis perhaps by targeting the MC-derived NOD-like receptor family pyrin domain containing 3 (NLRP3) inflammasome pathway and oxidative stress.

## 1. Introduction

Endometrium-like tissue that is present outside of the uterine cavity, particularly on the pelvic peritoneum and ovaries, is a symptom of the inflammatory disease called endometriosis, which is estrogen-dependent [1,2,3,4]. About 10% of women in their reproductive years experience it, and it is linked to pelvic pain and infertility [5]. Retrograde menstruation is generally accepted to be the source of ectopic endometrial tissue, and the peritoneal immunological milieu is critical for the progression of endometriosis. Inflammation in the peritoneal cavity is brought on by elements of refluxed blood, such as apoptotic endometrial tissue, desquamated menstrual cells, lysed erythrocytes, and released iron. Reactive oxygen species (ROS) released by triggered macrophages cause oxidative stress [5]. Mast cells (MCs) are crucial immune cell types whose function is disturbed in the peritoneal milieu of endometriosis [6]. Supplementary studies report that MCs play crucial roles in the development of endometriosis [7]. More and more evidence points to the involvement of MCs in inflammatory/autoimmune disorders as well by enhancing vascular permeability, facilitating immunological responses, and controlling fibrosis [8]. There are more activated MCs in endometriotic lesions, which may emit inflammatory mediators such as histamine and tumor necrosis factor (TNF)-α [9,10]. Additionally, after being exposed to stimuli, MCs can produce endogenous ROS [11]. An intracellular receptor called NLRP3 (NOD-like receptor family pyrin domain containing 3) detects both exogenous and endogenous danger cues [12] and interacts with apoptosis-associated speck-like protein (ASC), caspase-1 to construct the NLRP3 inflammasome complex. Interleukin (IL)-1β and other pro-inflammatory cytokines are released when the complex is activated, and it also stimulates immunological response and pyroptosis [13]. Prior research has also identified the special function of NLRP3 inflammasome in the activation of MCs during autoinflammatory conditions [14]. ROS is becoming a crucial regulator of inflammasome NLRP3 activation, which is connected to a number of illnesses [15]. Endometriosis progression is correlated to a pro-oxidative and immune mechanism and overproduction of ROS is associated with malignancy diffusion and increased proliferation rate [16]. Increased oxidative stress markers have been found in samples from women affected by this disease [1]. Oral contraceptives or nonsteroidal anti-inflammatory medicines are recommended as the first-line therapy for endometriosis, although many patients continue to exhibit pelvic and lesion size enlargement [3]. Interestingly, clinical studies supported that the administering antioxidants to women with endometriosis decreases their persistent pelvic pain and inflammatory indicators in peritoneal fluids [17]. Thus, the evaluation of therapeutic approaches targeting oxidative imbalance or related ROS molecular pathways could help to prevent endometriosis. Flavonoids are a large group of minor metabolites in plants containing over 4000 composites. Their principal subclasses are oxoflavonoids (flavonols and flavones), flavan-3-ol derivatives (such as tannins and catechin anthocyanins), and isoflavones [18]. Among flavonoids, the flavonol fisetin (3,3′,4′,7-tetrahydroxyflavone), is frequently present in fruits and vegetables. The largest concentration of fisetin was discovered in strawberries (160 μg/g) followed by apples (26.9 μg g/g) and persimmon (10.5 μg g/g) [19,20]. Fisetin is largely characterized by a diphenylpropane structure and contains two aromatic rings connected through three carbons oxygenated heterocyclic rings and united with one oxo and four hydroxyl groups as substituents. In particular, the quantity and location of the hydroxyl groups in this structure are closely related to its biological activities [21]. The biological effects of fisetin, such as anticancer [22,23] antidiabetic [24], anti-inflammatory [25], antioxidant [26], and neuroprotective effects [15,27] were investigated both in vitro and in vivo models [28]. Among these properties, fisetin looked to apply a solid cytotoxic action versus several varieties of cancers such as colon, lung, ovarian, liver, and breast [22,23]. In vivo tests on fisetin toxicity reported that the rats do not exhibit symptoms like decreased body weight, restlessness, respiratory discomfort, diarrhea, contractions, or coma [29,30]. Fisetin is considered a drug and nutritive supplement and is also recognized as a medical food, which stimulates more preclinical studies that could be translate into human clinical trials. One current clinical trial revealed important enhancement in anti-inflammatory markers when fisetin was injected into subjects with colorectal cancer [31,32]. A clinical study in stroke patients showed that fisetin was able to improve the treatment results, particularly in people with delayed onset-of-treatment [33]. Emerging findings indicated the possible health benefits of fisetin in aging as a senolytic through the lessening of senescent markers and age-related pathologies [34,35].

In that regard, a pilot study is also underway to examine the effectiveness of fisetin in decreasing inflammatory factors in the blood and in reducing fragility and markers of inflammation, insulin resistance, and bone resorption in elderly adults. ClinicalTrials.gov Identifier: NCT03675724.

Mechanistically, the anti-inflammatory activity of fisetin is often correlated with free radical scavenging and antioxidant properties, via regulating nuclear factor erythroid 2–related factor 2 Nrf-2/heme oxygenase-1 HO-1 and nuclear factor NF-κB, [26]. The previous study also reported that fisetin ameliorated inflammation and oxidative stress in lipopolysaccharide-induced endometritis [28]. Therefore, this study aimed to explore the antioxidant effect of fisetin on endometriosis considering the contribution of mast cells and the NLRP3 inflammasome pathway.

## 2. Results

### 2.1. Fisetin Ameliorates Endometriotic Lesions

**Figure 1:**
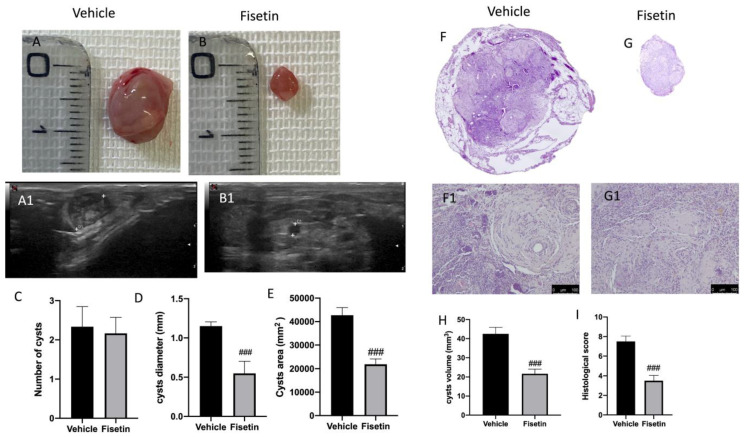
Fisetin administration on macroscopic and microscopic alterations: endometriosis + vehicle (**A**,**A1**), endometriosis + fisetin (**B**,**B1**), cyst number (**C**), cyst diameter (**D**), cyst area (**E**), cyst volume (**H**). Histological analysis of endometriosis + vehicle (**F**,**F1**), endometriosis + fisetin (**G**,**G1**), histological score (**I**). Figures are representative of at least three independent experiments. Values are means ± SEM of 6 animals for each group. Scale bar 100 µm. ^###^ *p* < 0.001 vs. vehicle.

At 14 days of induction—the end of the experiment—all animals from the vehicle and fisetin groups displayed endometriosis lesions (Figure 1A,B), while sham animals did not show any implants. Pelvic ultrasound evaluated the presence of endometriomas (Figure 1(A1,B1)). The evaluation included both anterior and posterior pelvic compartments to evaluate the different endometriosis locations (Figure 1(A1,B1)). The lesions from the fisetin group appeared smaller and more superficially attached to the peritoneal cavity compared to the vehicle group (Figure 1(B1)). These results were confirmed by macroscopic observation that showed that the cyst from the vehicle group was more evident than the cyst from the fisetin group (Figure 1A,B). In particular, both groups showed no distinctive number of cysts (Figure 1C). However, cysts diameter (Figure 1D), area (Figure 1E), and volume (Figure 1H) were lesser in the fisetin-treated group compared to the vehicle animals. Histologically, endometriotic lesions from vehicle-treated rats showed abundant stromal structure and endometrial-type glands (Figure 1(F,F1,I)). Fisetin administration reduced the histopathological marks of endometriosis (Figure 1(G,G1,I)).

### 2.2. Fisetin Reduces Mast Cell Activation on Endometriotic Lesions

**Figure 2:**
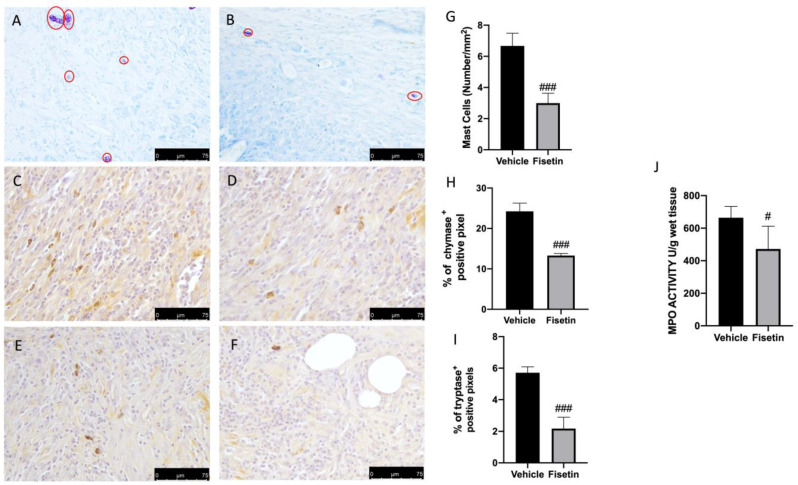
Fisetin administration on MCs activation: Blue toluidine staining to detect MCs (red circle) in endometriosis + vehicle (**A**), endometriosis + fisetin (**B**). Immunohistochemistry for chymase and tryptase in endometriosis + vehicle (**C**,**D**), in endometriosis + fisetin (**E**,**F**). Number of MC/mm^2^ (**G**). The results are expressed as % of positive pixels (**H**,**I**). Figures are representative of at least three independent experiments. MPO activity (**J**). Values are means ± SEM of 6 animals for each group. Scale bar: 75 μm. ^###^ *p* < 0.001 vs. vehicle ^#^ *p* < 0.05 vs. vehicle.

Several papers described the key role of inflammatory cell recruitment at the lesion site during endometriosis [36]. To better evaluate the role of MCs on endometriotic lesion, we performed toluidine blue staining. Explants from vehicle rats revealed increased MC recruitment (Figure 2A,G), while fisetin animals showed reduced MC infiltration (Figure 2B,G). In addition, to confirm the activity of MCs and their activation, we evaluated the chymase and tryptase expressions by immunohistochemical analysis. In particular, vehicle-treated animals showed increased positive staining for chymase and tryptase while the fisetin group showed reduced positivity (Figure 2C–I). Additionally, MPO a marker of neutrophil infiltration was also evaluated. Fisetin reduced MPO activity compared to the vehicle rats (Figure 2J).

### 2.3. Fisetin Reduces Fibrotic Process on Endometriotic Lesions

**Figure 3:**
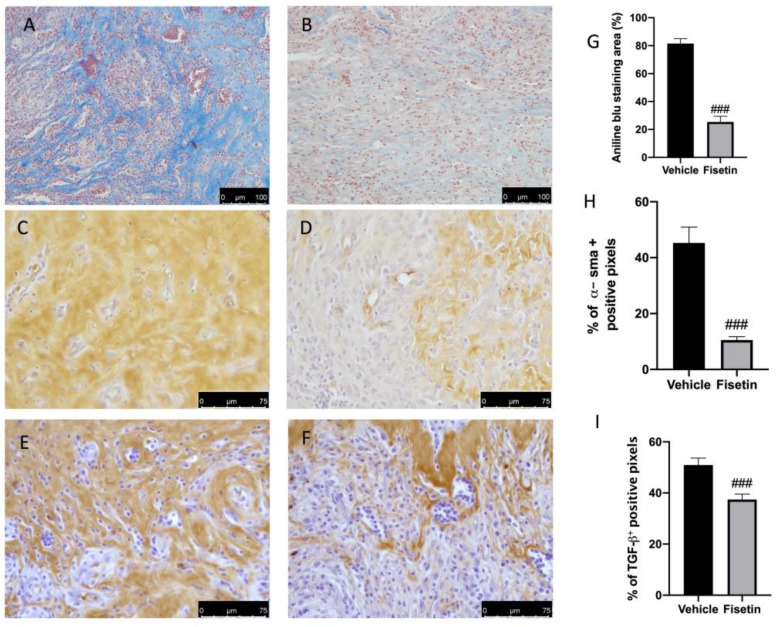
Fisetin administration on fibrosis: Masson trichrome staining for endometriosis + vehicle (**A**) +fisetin (**B**). Immunohistochemical analysis of α-sma and TGF-β for vehicle (**C**,**E**), fisetin (**D**,**F**). Aniline blue stain area (**G**); Immunohistochemical results are expressed as % of positive pixels (**H**,**I**). Figures are representative of at least three independent experiments. Values are means ± SEM of 6 animals for each group. Scale bar: 75 μm. ^###^ *p* < 0.001 vs. vehicle.

Fisetin administration also showed important anti-fibrotic effects. Masson trichrome staining showed a reduction in collagen fibers in lesions from fisetin rats (Figure 3B,G), compared to the vehicle (Figure 3A,G). In accordance with the staining, α-sma and TGF-β expressions were also evaluated. In particular, positive immunoreactivity was markedly increased in the vehicle group while the fisetin group showed reduced expression for α-sma and TGF-β (Figure 3C–I).

### 2.4. Fisetin Reduces Oxidative Stress on Endometriotic Lesions

**Figure 4:**
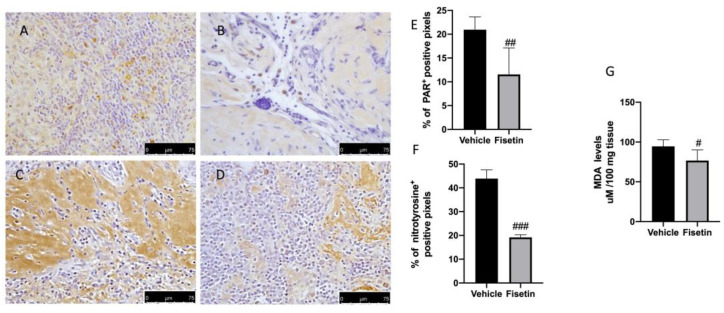
Fisetin administration on oxidative stress: Immunohistochemistry for PAR and nitrotyrosine in endometriosis + vehicle (**A**,**C**), in endometriosis + fisetin (**B**,**D**). The results are expressed as % of positive pixels (**E**,**F**). Figures are representative of at least three independent experiments. MDA levels (**G**). Values are means ± SEM of 6 animals for each group. Scale bar: 75 μm. ^###^ *p* < 0.001 vs. vehicle. ^#^ *p* < 0.05 vs. vehicle. ^##^ *p* < 0.01 vs. vehicle.

Several works demonstrated the role of ROS on endometriosis development [37]. Superoxide anion (O_2_**•**^−^) and nitric oxide (NO) can react with each other contributing to the formation of peroxynitrite (ONOO−). This molecule can then act on proteins, leading to the nitration of protein tyrosines by the formation of nitrotyrosine. In addition, upon DNA damage, poly (ADP-ribose) polymerase-1 (PARP-1) is activated and catalyzes the formation of poly(ADP-ribose) (PAR) chains by transferring (ADP-ribose) from NAD+ onto itself and nuclear acceptor proteins. In that regard, increased expression of nitrotyrosine (Figure 4C,F) and PAR (Figure 4A,E) were observed in the vehicle group compared to animals treated with fisetin (Figure 4B,D–F). MDA levels were also evaluated as markers of lipid peroxidation. MDA levels were increased in the vehicle group, while these levels were reduced by fisetin treatment (Figure 4G).

### 2.5. Fisetin Reduces Inflammasome Pathway and NF-κB Expression

**Figure 5:**
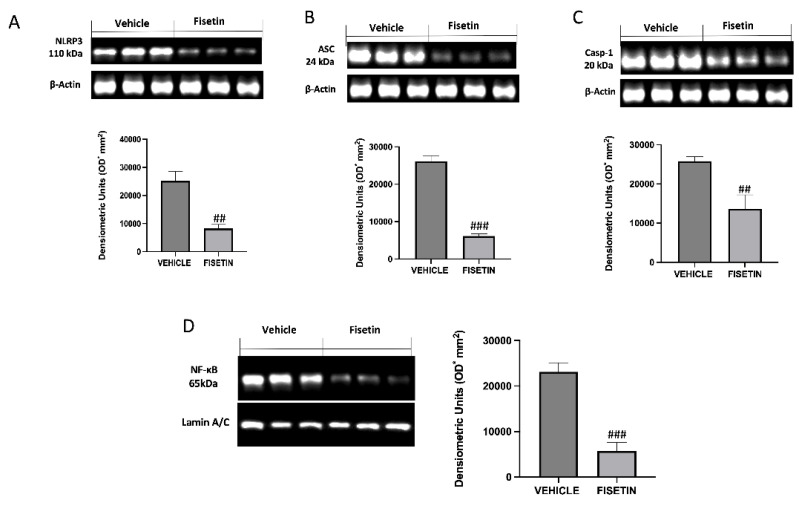
Fisetin administration on inflammasome activation and NF-κB. Western blots on endometriotic tissues for (**A**) NLRP3, (**B**) ASC, (**C**) caspase 1, (**D**) NF-κB p65. Exposed is a blot of lysates (6 animals/group) with a densitometric analysis for all animals. The results are means ± SEM of 6 animals for each group. ^###^ *p* < 0.001 vs. vehicle, ^##^ *p* < 0.01 vs. vehicle.

To better investigate whether fisetin could act by inhibiting the inflammasome pathway, we performed Western blots for NLRP-3, ASC, and cleaved caspase-1. The expression of the NLRP-3, ASC, and cleaved caspase-1 was significantly upregulated in endometriotic lesions tissues of vehicle rats (Figure 5A–C). Treatment with fisetin notably inhibited the NLRP3, ASC, and cleaved caspase-1 expression in endometriotic tissues of vehicle rats subjected to endometriosis (Figure 5A–C). In addition, increased nuclear NF-κB expression was observed in the vehicle rats subjected to endometriosis (Figure 5D). Fisetin significantly reduced the level of nuclear NF-κB compared to the vehicle group (Figure 5D).

### 2.6. Fisetin Reduces Proinflammatory Cytokines

**Figure 6:**
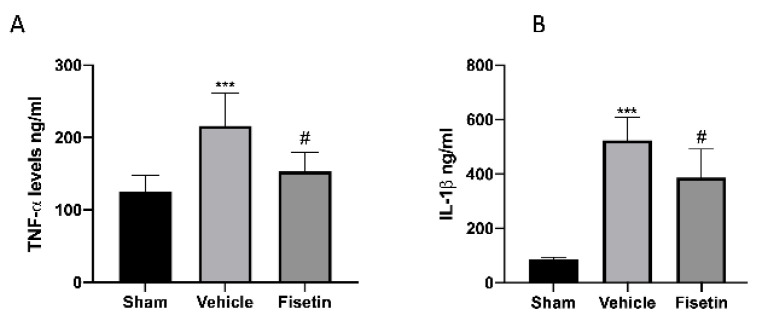
Fisetin administration on inflammatory cytokines in peritoneal fluids. TNF-α (**A**), IL-1β (**B**). The results are means ± SEM of 6 animals for each group. *** *p* < 0.001 vs. sham, ^#^ *p* < 0.05 vs. vehicle.

Several cytokines including IL-1β and TNF-α were reported to be increased in the PF of women with endometriosis [38]. In addition, the significance of inflammasome and successive excretion of IL-1 family members is supposed to be intricated in the pathogenesis of various diseases [39]. In that regard, increased levels of IL-1β and TNF-α were found in animals subjected to endometriosis and treated with the vehicle compared to the sham (Figure 6A,B). The oral administration of fisetin diminished these proinflammatory cytokines levels (Figure 6A,B).

### 2.7. Fisetin Increases Apoptotic Process

**Figure 7:**
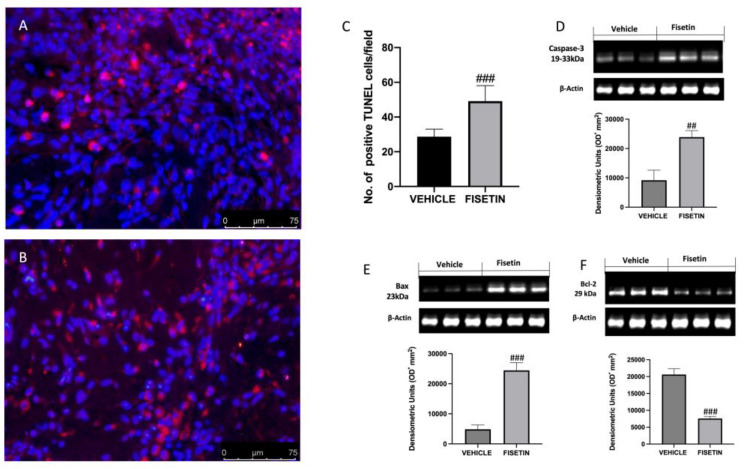
Fisetin administration on apoptosis: TUNEL staining to see positive apoptotic cells was performed in (**A**,**B**), endometriosis + vehicle (**A**) + fisetin (**B**). The number of apoptotic cells per field of endometriotic lesions (**C**). Figures are from at least three divided experiments. Western blots for caspase 3, Bax, and Bcl-2 (**D**–**F**). Exposed is a blot of lysates (6 animals/group) with a densitometric analysis for all animals. The results are means ± SEM of 6 animals for each group. ^###^ *p* < 0.001 vs. vehicle, ^##^ *p* < 0.01 vs. vehicle. Scale bar 75 μm.

The disparity between endometriotic cell growth and apoptosis characterized endometriosis. To research the effect of fisetin administration on apoptosis, TUNEL assay and Western blot analysis were conducted. To quantify cells undergoing apoptosis, the TUNEL assay was used. Tissue samples from animals displayed a reduced number of apoptotic cells (Figure 7A,C), while fisetin was able to increase the number of apoptotic cells (Figure 7B,C). The results were also confirmed by Western blot. Fisetin was able to increase apoptosis by reducing Bcl-2 and increasing Bax and caspase 3 levels (Figure 7D–F) with respect to vehicle rats.

## 3. Discussion

Women of reproductive age are susceptible to the gynecological and excruciating ailment known as endometriosis. It is characterized by non-uterine implants that resemble diseased endometrium. Although there is still debate on the pathophysiology of endometriosis, it is well-recognized that the inflammatory response is dangerous to this process. In this study, we provided mechanistic data on how fisetin, a natural flavonol, could have a positive action on estrogen-dependent endometriosis modulating MC-derived NLRP3 inflammasome.

Further studies report that MC activation by estrogen plays a crucial role in the development of endometriosis [7], through NLRP3 inflammasome activation [40,41]. Previous research suggested that estrogen can encourage the evolution of endometriotic lesions and have a role in the pathogenesis of endometriosis by stimulating MCs, which may increase the release of TNF-α and quicken the disease’s progression [42]. Targeted inhibition of NLRP3 considerably restrained lesion development and fibrogenesis in a mouse model of endometriosis [9]. Because erythrocytes, macrophages, and apoptotic endometrial tissues are well-known oxidative stress inducers, it is possible that the peritoneal generation of ROS contributes to endometriosis [37]. Various pieces of evidence support the role of oxidative stress in the progression of endometriosis [43,44]. This finding may pave the way for the assessment of therapeutic strategies targeting oxidative imbalance. Fisetin is one nutritive polyphenol that has been meticulously investigated [35,45]. It is extensively existed in a variety of vegetables and fruits, such as cucumbers, onions, strawberries, and apples. Animal studies have indicated that fisetin administration has favorable effects against diverse diseases [20]. It has been stated that fisetin has numerous biological activities, such as antioxidative, anti-inflammatory, and antitumor effects [46]. In vivo experimentations showed that fisetin lessened inflammatory damage and MPO activity in LPS-stimulated mouse endometritis [28]. Interestingly, the NF-κB inhibition or Nrf2 activation of fisetin has been determined in both rats and cells [47,48]. In addition, recent studies also confirmed that fisetin ameliorated several diseases by inhibiting inflammatory reactions via NLRP3, which is consistent with the results of the present study [49,50].

Based on these findings, in this study, we demonstrated that fisetin was able to decrease cyst diameter, area, and volume, histological alterations, neutrophil infiltration, the number of MCs, as well as chymase and tryptase expressions. Extensive adhesions and fibrosis present in progressive endometriotic lesions are linked to pelvic morbidities like chronic pelvic discomfort and infertility. Fisetin administration reduced collagen deposition, α-sma, and TGF-β expressions showing reduced fibrosis.

Oxidative stress induces ovarian injuries. In fact, endometriosis patients’ granulosa cells exhibit greater evidence of oxidative DNA damage. Granulosa cells of females with endometriosis display a higher prevalence of apoptotic bodies and nitrotyrosine than controls [51]. MDA has been valued as an index of lipid peroxides. Nasiri et al. also observed higher levels of MDA in the serum of subjects with endometriosis [52]. In that regard, here we evaluated markers of oxidative and nitrosative stress, in particular, we found high expression of nitrotyrosine and PAR expression as well as high levels of MDA in endometriotic lesions of vehicle rats while fisetin was able to decrease nitrotyrosine, PAR, and MDA levels. Additionally, it has been demonstrated that ROS play a significant role in triggering NLRP3 inflammasome [15]. NLRP3 inflammasome was initially designated by Tschopp et al. in 2002 [53] and is supposed to be indispensable in natural immunity [53]. The levels of NLRP3, caspase-1, apoptosis-associated speck-like protein, and IL-1 expression are considerably high during pathological conditions, causing an inflammatory response in the body, which, consequently leads to disproportionate production of several types of cell necrosis and programmed cell death [54]. Later, NLRP3 triggers a series of downstream signaling pathways, which determine the cleavage of inactive IL-1β and IL-18 precursors into mature and active IL-1β and IL-18, which are successively released into the extracellular compartment, instigating inflammatory responses and oxidative stress [49].

It has been suggested that NLRP3 inflammasome, which leads to the activation of IL-1β, contributes to the progression of endometriosis [55]. The activation of nuclear factor NF-κB, which increases the production of pro-inflammatory cytokines, is one of the detrimental effects of excessive ROS. NF-κB can incite the NLRP3 inflammasome and the maturation of pro-inflammatory cytokines [56]. Studies conducted in vivo and in vitro have shown its inflammatory activation in endometriotic cells [43]. ROS generation causes peritoneal macrophages to produce more NF-κB, which in turn causes endometriosis patients to produce proinflammatory, growth, and angiogenic factors [43]. Here, we demonstrated that endometriosis in rats caused the activation of NLRP3 and NF-κB pathways as well as the increase in levels of IL-1β and TNF-α, while fisetin inhibited the activation of NLRP3/NF-κB as well as diminished the levels of IL-1β and TNF-α. These results are in agreement with several works that showed that fisetin treatment was able to reduce the NF-κB/NLRP3 pathway as well as reduce the release of proinflammatory cytokines [15,57,58].

ROS generation is linked to enhanced proliferation rates in both tumor and endometriotic cells [59]. Apoptosis, which occurs during the menstrual cycle, keeps cells in a state of equilibrium by removing extra or malfunctioning cells. Apoptotic activity of endometriotic cells is regulated by diverse factors including both anti-apoptotic Bcl-2 and Bcl-xL and pro-apoptotic Bax and caspase-3. Several studies pointed out that the endometrium of endometriotic females is less sensitive to apoptosis than that of healthy controls [60,61]. In that regard, here we showed that rats subjected to endometriosis presented a reduced number of TUNEL-positive cells and reduced expression of proapoptotic proteins Bax and caspase 3 as well as increased Bcl-2. On the contrary, the administration of fisetin was able to increase the apoptotic process by increasing Bax and caspase 3 and reducing antiapoptotic Bcl-2 expressions.

In conclusion, in this study, we reported the relevance of MCs and the inflammasome pathway together with oxidative stress in the development of endometriosis. Fisetin administration was able to ameliorate oxidative damage induced by endometriosis in rats by reducing the activation of MCs and targeting the NF-κB/inflammasome pathway.

## 4. Materials and Methods

### 4.1. Animals

Sprague Dawley rats (Female, 250 gr) (Envigo, Milan, Italy) were housed in steel cages in a controlled room and were fed and watered regularly. The research was authorized by the University of Messina’s Animal Care Review Board. All animal tests complied with both Italian (D.Lgs 2014/26) and EU (EU Directive 2010/63) legislation.

### 4.2. Experimental Protocol

Endometriosis was established as already described [2]. Animals were randomly divided into two groups, donor or recipient. To stimulate similar estrogen levels, donor rats were intraperitoneally injected with 10 IU of pregnant mare serum gonadotropin and euthanized later. The uterus from donors was removed and minced with scissors. The recipient animals were injected intraperitoneally with the equivalent of tissue from one uterus in 500 uL of PBS (Sigma Aldrich, St. Louis, MO, USA) along the midventral line. It took seven days for endometriosis to clinically manifest with the endometrial cyst formation that was confirmed by abdominal high-frequency ultrasound analysis.

### 4.3. Experimental Groups

At 7 days after induction, rats were randomized and assigned to the following groups *n* = 12 for each group:

(1) Vehicle group: rats were subjected to the experimental procedure as described above, and vehicle (1% dimethyl sulfoxide solvent (DMSO, Sigma Aldrich, St. Louis, MO, USA) was orally administered by gavage, on the 7th day and for the next 7 days.

(2) Fisetin group: rats were subjected to an experimental procedure as described above, and fisetin (40 mg/Kg) was orally administered by gavage, on the 7th day and for the next 7 days.

(3) Sham group: rats were injected intraperitoneally with 500 uL of PBS without endometrial tissue, and vehicle (1% DMSO) was orally administered by gavage, on the 7th day and for the next 7 days.

The dose and route of administration of fisetin were chosen based on a previous study [15]. To evaluate the effect of fisetin on the endometriotic lesions, rats were sacrificed at 14 days after endometriosis induction. Laparotomy was performed to collect the endometriotic implants and peritoneal fluids and further processed for molecular analysis.

### 4.4. Histological Evaluation

Histological sections were stained with hematoxylin and eosin (H&E) and evaluated using a Leica DM6 microscope (Leica Microsystems SpA, Milan, Italy) associated with Leica LAS X Navigator software 3.4.2. Histopathologic scores were evaluated as described previously [4]. Additionally, lesion volume was calculated according to the formula: V = (length × width^2^) × 0.5 [62]. Mast cell evaluation was performed by toluidine blue staining [4].

### 4.5. Abdominal High-Frequency Ultrasound

At the 7th and 14th days post-induction, ultrasonographic examinations were performed using an Esaote MYLAB OMEGA VET (Esaote Italia, Milan, Italy) on sedated rats positioned in dorsal recumbency. Measurements were performed offline by a reader blinded to the condition of the rat as previously described [2,63].

### 4.6. Analysis of Myeloperoxidase (MPO) Activity

MPO activity was measured in endometriosis lesions as already described [43,44] and measured at 450 nm [64,65].

### 4.7. Cytokines Measurements

(TNF)-α and IL-1β and were determined using ELISA kits (BioLegend, San Diego, CA, USA; R&D Systems, Milan, Italy) in peritoneal fluids [66,67,68,69].

### 4.8. Immunohistochemical Analysis

Immunohistochemical analyses were performed in endometriosis lesions as already described [1,70,71,72,73]. The following primary antibodies were used: anti-α-sma antibody (Santa Cruz Biotechnology SCB,1:100 Biogenerica srl CT, Italy) and TGF-β (SCB, sc-130348 1:100) anti-chymase (1:100, SCB #sc59586 Biogenerica srl CT, Italy), and anti-tryptase (1:100, SCB, #sc59587 Biogenerica srl CT, Italy), anti-poly ADP ribose PAR (H-250: sc-7150, 1:500, SCB, Biogenerica srl CT, Italy), anti-nitrotyrosine antibody (Millipore, 06-284, Biogenerica srl CT, Italy), All sections were washed with PBS and then treated as previously reported [74]. Stained sections were observed using a Leica DM6 microscope (Leica Microsystems SpA, Milan, Italy). The histogram profile is related to the positive pixel intensity value obtained.

### 4.9. Apoptosis Tunel Assay

Apoptosis was analyzed by a TUNEL assay using a kit (DBA Italia, Milan, Italy) [65,75,76,77,78].

### 4.10. Western Blot Analysis

Cyst samples were homogenized, and Western blots were performed as already described [3,79,80,81,82,83,84]. The following primary antibodies were used: anti-nuclear factor NF-κB (SCB; 1:500 #sc8008, DBA Italia, Milan, Italy), anti-Bcl-2 (SCB, sc-7382, DBA Italia, Milan, Italy), anti-Bax (SCB, sc-7480, DBA Italia, Milan, Italy), anti-NRLP3 (SCB, sc-66846, DBA Italia, Milan, Italy), or anti-ASC antibody (SCB, N-15: sc-22514-R, DBA Italia, Milan, Italy), or anti-cleaved caspase 3 (sc-271028 SCB, DBA Italia, Milan, Italy), anti-Caspase-1 p20 (SCB, G-19: sc-1597 DBA Italia, Milan, Italy) in 1x PBS, 5% (*w/v*) non-fat dried milk, 0.1% Tween-20 at 4 °C overnight. Membranes were incubated with peroxidase-conjugated bovine anti-mouse IgG secondary antibody or peroxidase-conjugated goat anti-rabbit IgG (Jackson ImmunoResearch, West Grove, PA, USA; 1:2000, DBA Italia, Milan, Italy) for 1 h room temperature. Anti β actin or anti-lamin A/C (SCB, 1:5000, DBA Italia, Milan, Italy) antibodies were used as controls. The expression of protein bands was detected by a procedure previously described [3].

### 4.11. Materials

All chemicals were analytical grade or higher. Fisetin (Fustel) was purchased from Selleckchem, Biogenerica CT, Italy.

### 4.12. Statistical Evaluation

SEM = the mean standard error of the mean of N observations; N = the number of animals. The photos for histology/immunohistochemistry were from at least three different experiments. The results were analyzed by t-test when comparing two groups and one-way ANOVA followed by a Bonferroni post hoc test for multiple comparisons. The 0.05 *p*-value was taken as significant.

## Data Availability

The data presented in this study are available on request from the corresponding author.
